# Is it left or is it right? A classification approach for investigating hemispheric differences in low and high dimensionality

**DOI:** 10.1007/s00429-021-02418-1

**Published:** 2021-12-09

**Authors:** Patrick Friedrich, Kaustubh R. Patil, Lisa N. Mochalski, Xuan Li, Julia A. Camilleri, Jean-Philippe Kröll, Lisa Wiersch, Simon B. Eickhoff, Susanne Weis

**Affiliations:** 1 Institute of Neuroscience and Medicine, Brain and Behaviour (INM-7), Research Centre Jülich, 52428 Jülich, Germany; 2 Institute of Systems Neuroscience, Medical Faculty, Heinrich Heine University Düsseldorf, 40225 Düsseldorf, Germany

**Keywords:** Brain asymmetry, Functional laterality, Machine learning, Neuroimaging, Volumetry

## Abstract

Hemispheric asymmetries, i.e., differences between the two halves of the brain, have extensively been studied with respect to both structure and function. Commonly employed pairwise comparisons between left and right are suitable for finding differences between the hemispheres, but they come with several caveats when assessing multiple asymmetries. What is more, they are not designed for identifying the characterizing features of each hemisphere. Here, we present a novel data-driven framework—based on machine learning-based classification—for identifying the characterizing features that underlie hemispheric differences. Using voxel-based morphometry data from two different samples (*n* = 226, *n* = 216), we separated the hemispheres along the midline and used two different pipelines: First, for investigating global differences, we embedded the hemispheres into a two-dimensional space and applied a classifier to assess if the hemispheres are distinguishable in their low-dimensional representation. Second, to investigate which voxels show systematic hemispheric differences, we employed two classification approaches promoting feature selection in high dimensions. The two hemispheres were accurately classifiable in both their low-dimensional (accuracies: dataset 1 = 0.838; dataset 2 = 0.850) and high-dimensional (accuracies: dataset 1 = 0.966; dataset 2 = 0.959) representations. In low dimensions, classification of the right hemisphere showed higher precision (dataset 1 = 0.862; dataset 2 = 0.894) compared to the left hemisphere (dataset 1 = 0.818; dataset 2 = 0.816). A feature selection algorithm in the high-dimensional analysis identified voxels that most contribute to accurate classification. In addition, the map of contributing voxels showed a better overlap with moderate to highly lateralized voxels, whereas conventional *t* test with threshold-free cluster enhancement best resembled the LQ map at lower thresholds. Both the low- and high-dimensional classifiers were capable of identifying the hemispheres in subsamples of the datasets, such as males, females, right-handed, or non-right-handed participants. Our study indicates that hemisphere classification is capable of identifying the hemisphere in their low- and high-dimensional representation as well as delineating brain asymmetries. The concept of hemisphere classifiability thus allows a change in perspective, from asking what differs between the hemispheres towards focusing on the features needed to identify the left and right hemispheres. Taking this perspective on hemispheric differences may contribute to our understanding of what makes each hemisphere special.

**Supplementary Information:**

The online version contains supplementary material available at 10.1007/s00429-021-02418-1.

## Introduction

One of the most fundamental ways of gaining a deeper understanding of the brain is to study the differences between its constituting parts. Comparing the two halves of the brain has identified lateralization of functions as a core phenomenon of brain organization, spanning across various cognitive domains. The leftward processing asymmetry for speech perception and production (Van der Haegen et al. 2013; Hugdahl and Westerhausen 2016), semantic word processing (Koppehele-Gossel et al. 2018; Hoffman et al. 2018), and hand motoric (Amunts et al. 2010; Guadalupe et al. 2014), as well as the right-hemispheric dominance for visuospatial attention (Thiebaut de Schotten et al. 2011; Zago et al. 2017) and face perception (Dundas et al. 2013; Adibpour et al. 2018), are among the most common examples of functional lateralization in humans. In addition, the associations between atypical functional laterality and various neurological and psychiatric illnesses such as Alzheimer’s Disease (Thompson et al. 1988), autism (Floris et al. 2020; Jouravlev et al. 2020), ADHD (Chan et al. 2009) and schizophrenia (Hirnstein and Hugdahl 2014) underline the importance of understanding the neural underpinnings that may drive functional hemispheric asymmetries. However, focusing on the differences alone may not yield a comprehensive characterization of the brains constituting parts.

In general, functional laterality is accompanied by structural asymmetries in gray and white matter (Ocklenburg et al. 2016), which are observable on the macroscopic and microscopic levels (Amunts 2010). For instance, MRI studies have identified volumetric asymmetries in brain regions within the language network, including the planum temporale (Lyttelton et al. 2009; Luders et al. 2004; Geschwind and Levitsky 1968), superior and middle temporal gyrus as well as the constituting cortices of Broca’s area (Kong et al. 2018). However, there is no consensus on hemispheric asymmetries across modalities and regions. For instance, some studies support a leftward asymmetry of Broca’s area, both insurface (Falzi et al. 1982) as well as with regard to cytoarchitectonics (Amunts et al. 1999), whereas other studies were unable to find convincing leftward asymmetries (See Keller et al. 2009, for review). While differences in these studies may be due to small sample sizes and potential influences of phenotypes such as sex, age and handedness (Guadalupe et al. 2014), more recent studies have investigated asymmetries using bigger samples (Kong et al. 2018; Chiarello et al. 2016; Koelkebeck et al. 2014; Plessen et al. 2014; Zhou et al. 2013; Maingault et al. 2016), but still show inconsistencies.

The lack of consensus across studies may also be driven by a variety of methodological differences. As highlighted by Chiarello et al. (2016), two different strategies are typically applied for examining brain asymmetries. On the one hand, asymmetries can be investigated based on a point-by-point comparison of the left and right-hemispheric surfaces after determining corresponding points (Luders et al. 2006; Plessen et al. 2014; Van Essen et al. 2012). On the other hand, cortical asymmetries can also be expressed based on a region-by-region comparison, which relies on the definition of corresponding brain regions (Koelkebeck et al. 2014). Both of these strategies come with several limitations. While surface-based analyses are well suited for analyzing microstructure, activation patterns and functional connectivity (Fukutomi et al. 2019; Brodoehl et al. 2020) of gray matter, they are not applicable to the investigation of white matter tracts. In contrast, region-wise analyses in volume space are fundamentally dependent on the chosen parcellation of the brain in either gray or white matter. There is a range of potential atlases to choose from, encompassing different modality-specific properties including macroscopic anatomical information such as sulcal-boundaries (Tzourio-Mazoyer et al. 2002; Desikan et al. 2006; Destrieux et al. 2010), cytoarchitecture (Eickhoff et al. 2005; Zilles and Amunts 2010) functional connectivity (Gordon et al. 2016; Schaefer et al. 2018; Yeo et al. 2011, Joliot et al. 2015, Shen et al. 2013), or a combination of multiple modalities (Glasser et al. 2016; Fan et al. 2016). As these atlases are quite diverse and optimized for different applications, results may vary between studies that use different parcellation schemes. Additionally, and regardless of the chosen strategy, the statistical results ought to be corrected for multiple comparisons if the aim is to investigate asymmetries across various structural entities (areas, tracts, nodes) within the same sample. Hemispheric asymmetries, however, typically show small effect sizes which makes it particularly difficult to find consensus across studies with differing samples sizes. This issue of sample size and statistical power is especially relevant for studies focusing on group comparisons, such as clinical cohorts versus healthy controls. In general, the majority of studies (both in healthy and clinical populations) circumvents the lack of power by reducing the focus to few specific anatomical entities (Roiser et al. 2016). However, limiting the search space, by definition, can not give the full picture of the investigated phenomenon.

Besides these methodological considerations, the goal of studying univariate asymmetries lies in the identification of local differences between the two hemispheres. Outside of this local search, some studies have shifted their attention towards more global architectural differences between the two hemispheres which is typically achieved based on other brain representations. As defined by Bijsterbosch et al. 2020, brain representations typically comprise a set of units (e.g., parcels, nodes) and a summary measure (e.g., pairwise correlation) that is applied at the level of these units. For instance, graph theory has been used to find hemispheric differences in the topology of unilateral functional networks (Cao et al. 2020; Tian et al. 2011) as well as in hemispheric white matter connectivity (Caeyenberghs and Leemans 2014; Itturia-Medina et al. 2011). These studies point to more general differences in the organization of the two hemispheres that are affected by both gender and age (Sun et al. 2017; Tian et al. 2011). Convergently, they indicate that the left hemisphere is more strongly organized around indispensable hub regions, which makes it especially suitable for high-demand and specific tasks, whereas the right hemisphere displays a more distributed organization, potentially allowing stronger focus on integration and general tasks. In accordance, a study that characterizes intra- and inter-hemispheric resting-state connectivity indicated a higher tendency for left hemispheric areas involved in language and motor functions to interact unilaterally, whereas right-hemispheric regions involved in visuospatial attention displayed higher bilateral interaction (Gotts et al. 2013). Brain representations that assess global organization principles are typically based on decomposing data from distinct brain units (e.g., areas, voxels, and nodes) to represent the common variance among them. Prominent examples of this strategy revealed the existence of functional networks using principal component analyses (Smith et al. 2009) in the resting brain or a principal gradient shown by manifold learning (Margulies et al. 2016), which may represent an organizational axis of the brain’s functional topology (Huntenburg et al. 2018). Regarding hemispheric differences, recent studies indicate meaningful characterizations of the two hemispheres based on dimensionality reduction. For instance, low-dimensional embedding of lateralized functions revealed the taxonomy of functional laterality comprises four functional domains, including symbolic communication, perception/action, emotion, and decision making (Karolis et al. 2019). With regard to resting-state connectivity, another recent study showed asymmetries in the functional gradients of the left and right hemispheres (Liang et al. 2021), thus indicating a more general difference in the functional hierarchy of the two hemispheres, which was not identifiable based on regional analyses alone. Conceptually, approaches for investigating hemispheric differences on a more global architectural scale are based on reducing the dimensionality of the underlying hemispheric metric either by means of a summary metric of the feature of interest or by decomposition of the high-dimensional data. As these approaches have qualitatively different results compared to parcellation-based or voxel-wise univariate comparisons, a complete understanding of two hemispheres likely needs both the low- and high-dimensional perspectives.

While high-dimensional, parcellation-based studies and low-dimensional, topology-based studies have shed light onto hemispheric differences, we are yet left with an inconclusive portrayal of the left and right hemispheres. Past research has mostly targeted the differences between homotopic parts of the two hemispheres, whereas the study of asymmetries should rather help to understand what makes each hemisphere special. Despite the importance of mapping and understanding asymmetries, the focus on differences alone does not grant a full characterization of either one of the two hemispheres. It is, therefore, still an open question which features, or properties of either hemisphere are defining characteristics that allow us to distinguish the hemispheres from one another. However, statistical comparisons, which are geared towards finding differences are by definition not designed to answer this type of question. Hence, the search for the determining characteristics that allow for differentiating the two hemispheres implies both a change of method and perspective. Instead of conventional statistical comparisons, machine learning is used to classify data points within a given dataset, based on a set of features. Machine learning-based classification approaches have been successfully applied to distinguish between males and females (Weis et al. 2020). Classification approaches are also applied to distinguish healthy controls from patient groups such as for example ADHD (Peng et al. 2013), Schizophrenia (Cai et al. 2020), or Alzheimer’s disease (Klöppel et al. 2008). Furthermore, tools such as feature selection can tell us which features that were fed into the classifier were especially important for the identification of group belongingness. In comparison with conventional statistical comparisons, machine learning-based classification presents a multivariate method instead of univariate analysis. Thus, machine learning-based classification and feature selection can be useful to learn more about the determining features from a multivariate standpoint. Therefore, this approach allows us to evaluate which features are relevant within a multivariate framework, whereas univariate statistical comparisons reveal which features significantly differ between two sets of observations (e.g., participant groups or hemispheres). As such, machine learning-based classification represents a complementary tool that can limit the search space for conventional statistical comparisons. However, so far, applications of machine learning in neuroscience have mostly been employed on the level of individual participants but have not yet been applied to the study of hemispheric differences within participants.

In this study, we present a novel approach for investigating hemispheric differences which appears suitable to decrease the searchspace for finding hemisphere-defining characteristics. In contrast to univariate statistical comparisons which aim to find features that differ significantly between the hemispheres, we shift the focus towards finding the features that are particularly important for correctly identifying a hemisphere as either left or right. This adaptation of the central question about hemispheric asymmetries calls for a classification approach, which may be well suited to find the determining characteristics of either hemisphere within the chosen brain representation or modality of interest, and thus approaching a better understanding about what makes each hemisphere special. As a proof-of-concept of the proposed classification approach, we investigate the classifiability of each hemisphere based on their volumetry in their low-dimensional representation (via manifold learning) as well as in their high-dimensional (voxel-wise) representation.

## Methods

### Dataset

We used preprocessed VBM-images of two open access datasets from the Amsterdam Open MRI Collection (AOMIC; for details see Snoek et al. 2021). The two datasets (dataset 1 = PIOP1; dataset 2 = PIOP2) consist of 226 and, respectively, 216 participants (dataset 1: age = 22.18 ± 1.8, females/males/unknown = 120/89/7; dataset 2: age = 21.96 ± 1.79, females/males/unknown = 129/96/1). For both datasets, handedness was acquired via self-report. Dataset 1 consists of 180 right-handed, 24 left-handed and 5 ambidextrous participants. Dataset 2 consists of 201 right-handed, 22 left-handed and 1 ambidextrous participants. The datasets are openly available via openneuro.org (for more details see: https://nilab-uva.github.io/AOMIC.github.io/). Two distinct datasets were used to ensure that the results are not dependent on the specific dataset. While these two datasets share the same acquisition protocols, contrast-to-noise ratio is improved in dataset 2, due to an improvement of scanner hardware in between acquisition of the two datasets (Snoek et al. 2021).

### T1 MRI acquisition and VBM preprocessing

T1-weighted anatomical images were acquired for co-registration and voxel-based morphometry analyses, with both datasets using the same acquisition parameters. Anatomical images were obtained from a 3D MPRAGE sequence with 2 repetitions (FOV = 188*240*200, TR/TE = 8.5/3.9, bandwidth = 191.5 Hz/pix, flip angle = 8°, SENSE-factor = 2.5 RL/2 FH) in axial acquisition direction. The total scan duration was 6:03 min.

Voxelwise gray matter volume maps were derived from voxel-based morphometry analyses using FSL-VBM (http://fsl.fmrib.ox.ac.uk/fsl/fslwiki/FSLVBM; Dounaud et al. 2007). The protocol was optimized in accordance with Good et al. (2001) and used a combination of FSL Tools (Smith et al. 2004) and fMRIPrep (Esteban et al. 2019). Here, fMRIPrep yielded probabilistic gray matter segmentation for each participant in native space, which allows skipping initial preprocessing steps of the standard FSL-VBM pipeline, including brain extraction and segmentation. The probabilistic gray matter segmentation was registered to the MNI152 standard space via non-linear registration. Resulting images were averaged across participants and flipped along the X-axis to create a study specific left–right template. Subsequently, all native gray matter images were non-linearly registered to the resulting template and corrected for local expansion caused by the non-linear spatial transformation. Aligning onto the symmetrical template was done to allow for spatial comparisons between the two hemispheres.

### Study-specific processing of the VBM derivatives

**Fig. 1:**
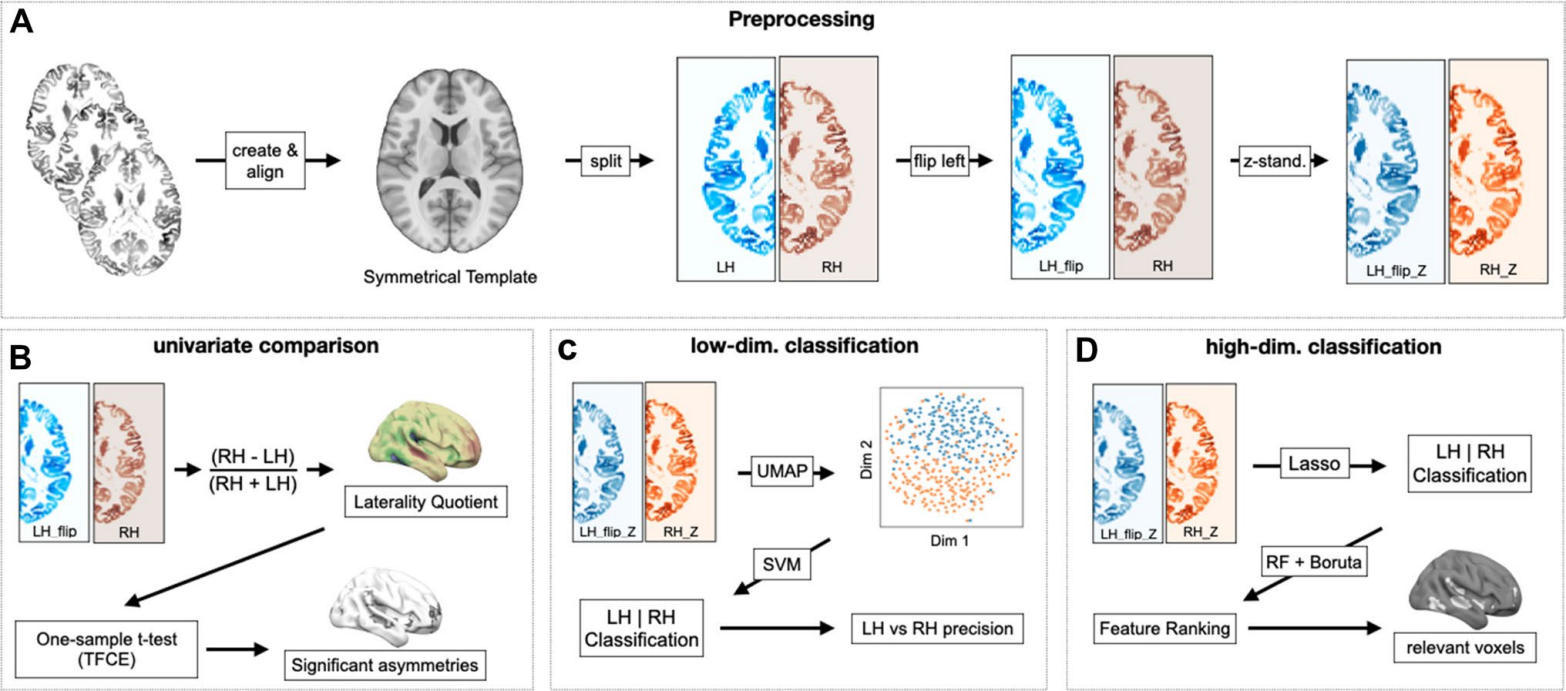
Methodological overview. **A** Processing after creating the measurement of interest (in this case VBM values). Images were aligned onto a symmetrical, sample-specific template. The hemispheres were split, aligned and z-standardized. **B** Processing steps for conventional statistical comparison. Two outcomes were generated: a laterality quotient image was created which represents the averaged vbm asymmetry per voxel. Significant asymmetries were accessed via demeaned one-sample T-test with threshold-free cluster enhancement. **C** Processing steps for low-dimensional classification. Dimensionality of all hemispheres was reduced via UMAP. The low-dimensional representation of each hemisphere was fed into a support vector machine to classify hemispheres as left or right. We assessed the precision for classifying the left and right hemispheres based on their low-dimensional representation. **D** Processing steps for high-dimensional classification. Voxels of the left and right hemispheres were fed into a LASSO classifier, which gave the classification accuracy for each hemisphere as left or right on the basis of selected features. The Boruta feature selection algorithm was applied based on a random forest classifier, to identify the voxels that were most informative for correct classification of a given hemisphere

An overview of the processing steps is presented in Fig. 1 and all depicted steps were applied to both datasets. We split the left and right hemispheres along the midline and flipped the left hemisphere across the X-axis to align the left and right hemispheres (Fig. 1A). Flipping the left hemisphere “onto” the right hemisphere aims to make the voxel indices comparable between them. Thus, all voxels in one hemisphere directly match the same position in the other hemisphere. Subsequently, we conducted three distinct lines of analyses for finding hemispheric asymmetries including a univariate voxel-wise comparison (Fig. 1B); classification of the hemispheres based on their low-dimensional representation (Fig. 1C); and classification of the hemispheres based on voxel-wise information (Fig. 1D). First, we created a voxel-wise laterality quotient (LQ) by subtracting the left hemisphere from the right hemisphere for each participant and dividing the results by the sum of left- and right-hemispheric VBM values. The resulting LQ images were concatenated to create a 4D image per dataset. The average laterality quotient map was used to compare results from other approaches with the sample-averaged asymmetries in volumetry. For the conventional univariate comparison approach, we assessed the significance of voxel-wise asymmetries via a one-sample *t* test, with threshold-free cluster enhancement (TFCE) using FSL randomise with 5000 permutations. Results of the *t* tests are presented in “High-dimensional analyses: univariate comparisons”.

All machine-learning analyses were based on the scikit-learn package (Pedregosa et al. 2011) and all matrix operations were conducted in the python 3.8 environment. Prior to low- or high-dimensional classification, all hemispheres were read as 3D matrices using ‘nilearn’. Matrices were transformed to one-dimensional vectors with length V, where V is the number of voxels. The resulting vectors of shape 1*V were concatenated to create a N*V data matrix, where N is the number of hemispheres (sample size * 2; Dataset 1: *N* = 432; Dataset 2: *N* = 452). Given that the average volumetry between the left and right hemispheres differs (Kong et al. 2018), values within each hemisphere were standardized using Z-score. This step was designed to prevent classifiers from only using the hemispheric difference in averaged volumetry for identifying the hemispheres.

### Statistical analyses and machine learning

To identify differences in volumetry between the left and right hemispheres, we calculated one-sample *t* tests on the LQ images with 0 as reference, using FSL randomise with TFCE. Results are reported with corrected *p* value < 0.05 (see Fig. 3A).

Given that properties of the brain can be represented with different granularity, we chose to base our classification analyses on the lowest and most high-dimensional representations possible. Thus, for the low-dimensional representation, we reduced dimensionality as much as possible, i.e., to two dimensions. On the other hand, we also tested the classifiability of the two hemispheres in their high-dimensional representation, in which each voxel is treated as an individual feature of interest. For both classification approaches, the data of a given dataset was presented as a N*V datamatrix, with *N* = number of participants * 2 (because each hemisphere is represented independently) and *V* = the number of voxels.

In the low-dimensional classification approach, we first reduced dimensionality of the data matrix using uniform manifold approximation and projection (UMAP; McInnes et al. 2018). UMAP is a manifold learning technique that is applicable for dimensionality reduction, which shares similarities to t-SNE (Van der Maaten and Hinton 2008) but preserves more of the global, topological data structure. In short, UMAP preserves local neighborhoods in a given dataset and presents distances as a weighted graph. Here, the distance between data points (hemispheres) in a lower dimensional space depends on the similarity/dissimilarity across the original dimensions (voxels), with higher proximity between hemispheres that are similar and higher distance between hemispheres that are dissimilar when taking all voxels into account. We chose to reduce dimensionality of the hemispheres to two dimensions, to better visualize and explore the low-dimensional representation of the hemispheres. To classify the hemispheres in low-dimensional space, a support vector machine (SVM) with a linear kernel was applied. An SVM approach was chosen here, as with only two dimensions, sparseness of the classifier is of no relevance. For each dataset, we used cross-validation (CV) using the default of 5-folds and 5-repetitions as implemented in JuLearn (https://juaml.github.io/julearn/main/index.html) to determine the accuracy for hemisphere classification. The results of this approach are reported in “Low-dimensional classification”.

In the high-dimensional classification approach, we used the “least absolute shrinkage and selection operator” (LASSO) algorithm with cross-validation using the default of threefold and one repetition. Here, all voxels of a given hemisphere were treated as input features. LASSO promotes sparseness by shrinking the weights of the irrelevant features—i.e., voxels—to zero. However, it is susceptible to multicollinearity and can ignore some features that are asymmetric. To address this limitation of LASSO, we additionally also employed the Boruta feature selection algorithm which is designed to identify “all relevant” features (Kursa and Rudnicki 2010). Results of the Boruta feature selection were reshaped from 1D into 3D space for subsequent visualization on a mesh via SurfIce (https://www.nitrc.org/projects/surfice/), and reported in “High-dimensional analyses: classification”.

For both the *t* test and Boruta selection, we used FSL’s cluster algorithm combined with the “atlas query” command to identify the most likely regions depicted by the respective result-map. To compare the results gained from the *t* test and Boruta selection, we computed a dice similarity coefficient (DSC) between the leftward (negative LQ) or rightward (positive LQ) asymmetry maps across different thresholds in increments of 0.2 with range from 0.2 to 6 for the positive LQ values, and −0.2 to −6 for the negative LQ values. This comparison is reported in “Comparing LQ, *t* test and Boruta selection via dice similarity coefficient”.

In addition to the outlined procedure, we also tested the hemisphere classifiability in low- and high-dimensional space separately for the two sexes as well as for n right- and non-right-handed participants. In low-dimensional space, we tested the classifiability of the two hemispheres as outlined above using UMAP and subsequent SVM. In high-dimensional space, LASSO was used to evaluate the classifiability and the overlap between males and females, or respectively, right- and non-right-handed participants were assessed via the DSC. This line of analyses is reported in “Hemisphere classifiability across sex and handedness”.

## Results

### Low-dimensional classification

**Fig. 2:**
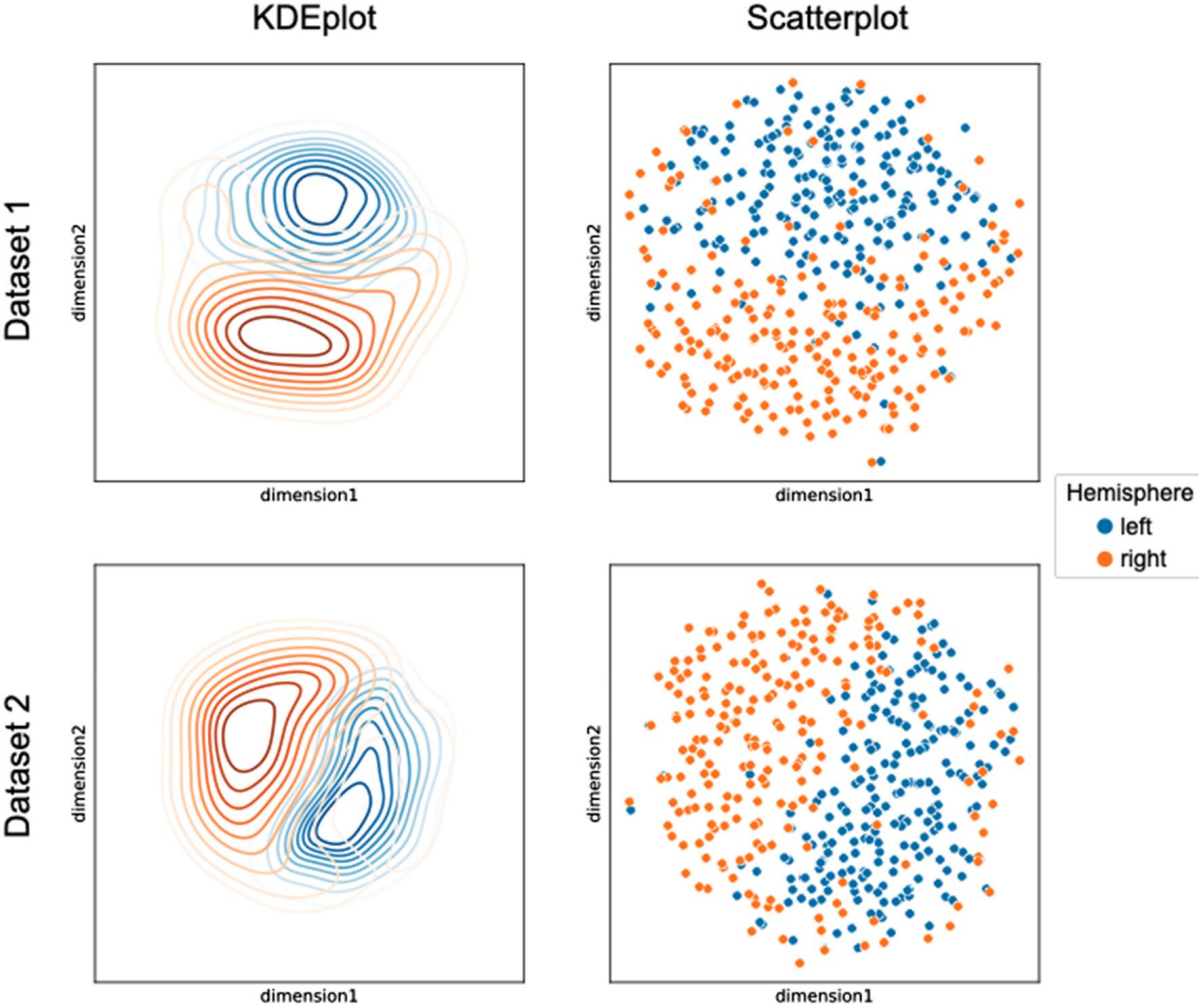
Low-dimensional embedding of the left and right hemispheres. Kernel-density estimate plots are visualized in the left column, showing the probability density of the left (blue) and right (orange) hemispheric 2-dimensional representation. Scatterplots in the right column show the distribution of the left and right hemispheres in two dimensions. Results of dataset 1 are depicted on the upper panel and results of dataset 2 are depicted in the lower panel

The dimensions of voxel-wise representations of the left and right hemispheres were reduced to two, via joint-embedding of the two hemispheres using UMAP. As outlined above, UMAP preserves the local neighborhoods, thus hemispheres displaying high similarities across voxels are depicted more closely to one another. Figure 2 depicts the low-dimensional representations, with the left side of the image showing the KDE (kernel-density estimate) plot and the right side depicting the corresponding scatterplots. As can be seen from the images, for both datasets, left hemispheres show a smaller spread compared to the right hemisphere. While the set of all hemispheres do not fall cleanly into two spatially separated clusters, the overall placement of hemispheres in 2D is not random. Both left and right hemispheres tend to group more closely with other hemispheres from the same side. This is particularly the case for left hemispheres, which shows a more compact probability density compared to right hemispheres. In accordance, F-tests revealed higher variance in the spreading of right compared to the left hemispheres in either the first (dataset 2: F(1) = 18.706, *p* < 0.001) or the second dimension (dataset 1: *F*_(1)_ = 18.275, *p* < 0.001) in both datasets.

Despite the lack of complete spatial separation between the left and right hemispheres in the two-dimensional embedding, the systematic positioning of hemispheres suggests testing their classifiability in this low-dimensional representation. Feeding the support vector machine with the dimension scores as input data and the side label (left/right) as target indicates high accuracy for hemispheric classification with averaged cross-validation (CV) accuracy = 0.838 in dataset 1 and averaged CV accuracy = 0.850 in dataset 2. The accuracies across CV runs range from 0.802 to 0.897 in dataset 1, and 0.8 to 0.9 in dataset 2. Accuracy of hemisphere classification did not differ significantly between the two datasets as indicated by an independent sample *t* test (*t*_(48)_ = − 1.593, *p* = 0.118).

We assessed the precision for correctly classifying left and right hemispheres in each dataset. For dataset 1, left hemispheres were correctly identified with a minimum precision of 0.761 and a maximum precision of 0.875. The mean precision across CV runs was 0.818. In comparison, precision for correctly classifying the right hemispheres ranged from 0.744 to 0.972 with a mean precision of 0.862. For dataset 2, precision for correctly identifying left hemispheres ranged from 0.72 to 0.887, with a mean precision of 0.816. Precision for classifying the right hemispheres were higher with a minimum precision of 0.767, maximum precision of 1.0 and an averaged precision of 0.894. An independent sample *t* test indicated that precision for correctly classifying right hemispheres was significantly higher compared to precision for classifying left hemispheres (dataset 1: *t*_(48)_ = − 3.515, *p* < 0.001; dataset 2: *t*_(48)_ = − 5.749, *p* > 0.001).

### High-dimensional analyses: univariate comparisons

To identify significant differences in VBM values between the left and right hemispheres, we used a one-sample *t* test on the laterality quotient maps corrected with threshold-free cluster enhancement (TFCE). Notably, given that TFCE corrected *t* test was used on the voxel-level, this *t* test did not provide a clear leftward or rightward asymmetry on a regional level. Using FSL’s atlas query indicated the presence of both leftward and rightward asymmetric voxels in various regions defined as defined by the Harvard–Oxford atlas including the frontal pole, insular cortex, superior frontal gyrus, middle frontal gyrus, inferior frontal gyrus, precentral gyrus, superior temporal gyrus, middle temporal gyrus, inferior temporal gyrus, postcentral gyrus, superior parietal lobule, supramarginal gyrus, angular gyrus, lateral occipital cortex, intracalcarine cortex, frontal medial cortex, supplementary motor cortex, subcallosal cortex, paracingulate gyrus, cingulate gyrus, precuneus cortex, cuneal cortex, frontal orbital cortex, parahippocampal gyrus, lingual gyrus, temporal fusiform cortex, and temporal occipital fusiform cortex.

### High-dimensional analyses: classification

**Fig. 3:**
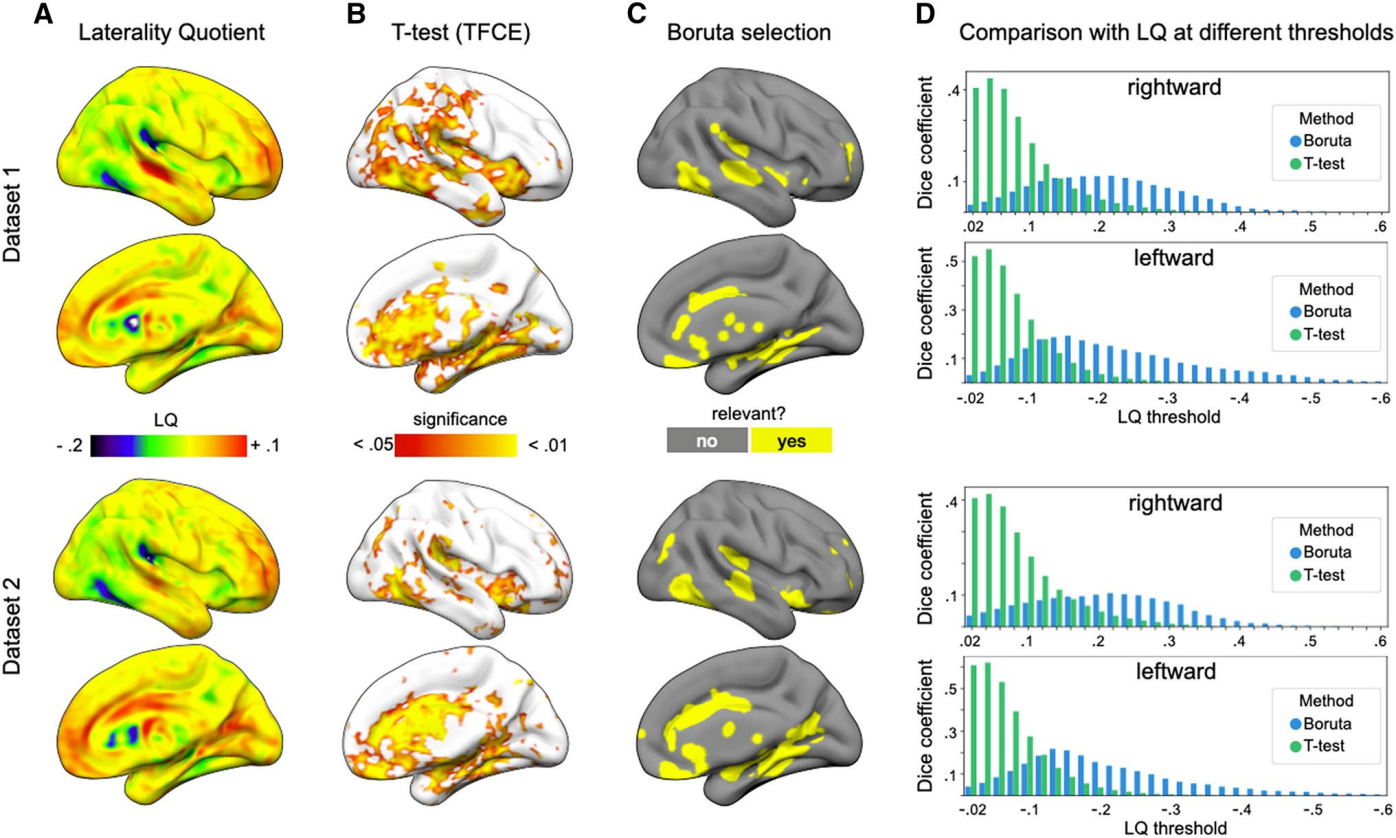
Comparing laterality quotient, *t* test and Boruta feature selection. All images are depicted on the right hemispheres. Results of dataset 1 are depicted on the upper panel and results of dataset 2 are depicted in the lower panel. **A** Laterality quotient. Positive values indicate rightward asymmetry and negative values indicate leftward asymmetry. **B** Significant voxels with* p* value below 0.05 corrected with TFCE. **C** Boruta selection. Yellow voxels were chosen as relevant features for distinguishing between the left and right hemispheres as a result of the cross-validation process. **D** Comparison between LQ maps with either the* t* test results or Boruta selection based on the dice similarity coefficient at different LQ thresholds. Dice similarity coefficients (y-axis) are shown for Boruta selection (blue) and *t* test (green) at different LQ thresholds (x-axis) for LQ maps of rightward (upper panel) and leftward (lower panel) voxels

The accuracy of voxel-based hemispheric classification was assessed using a LASSO classifier. For dataset 1, cross-validation accuracy ranged from 0.583 to 0.993, with mean accuracy of 0.966. Similarly, cross-validation accuracy for dataset 2 ranged from 0.668 to 0.985, with mean accuracy of 0.959. Results were comparable between the two datasets, indicated by an independent sample *t* test (t_(157)_ = 0.814, *p* = 0.417). As LASSO promotes sparse results, non-zero feature weights will identify voxels that contribute most strongly to high prediction accuracy. However, LASSO, by design, will ignore features that are (highly) correlated with each other and thus will not identify all features/voxels displaying relevant differences between the hemispheres. To delineate those, we employed the Boruta feature selection method that can uncover “all-relevant” features (Fig. 3C). FSL’s atlas query indicated that most voxels chosen by Boruta resided within regions including the insular cortex, superior temporal gyrus, middle temporal gyrus, inferior temporal gyrus, supramarginal gyrus, lateral occipital cortex, frontal medial cortex, subcallosal cortex, cingulate gyrus, frontal orbital cortex, parahippocampal gyrus, lingual gyrus, temporal fusiform cortex, fusiform cortex, occipital fusiform gyrus, central opercular cortex, parietal operculum cortex; Heschl’s gyrus, and prominently the planum temporale across both datasets. In addition, dataset 2 also shows some clusters of voxels in the frontal pole, paracingulate gyrus, precuneus, cuneal cortex, and the supracalcarine cortex.

### Comparing LQ,* t* test and Boruta selection via dice similarity coefficient

The similarity between the different maps was assessed using a dice similarity coefficient. Comparing the result-maps of the TFCE corrected *t* tests with the Boruta-selected voxels indicated some small similarities between the two methods in both datasets (dataset 1: DSC = 0.060; dataset 2: DSC = 0.070), due to more voxels reaching the significance threshold in the *t* tests (Fig. 3).

We furthermore investigated the similarity of both methods with the laterality quotient across different laterality thresholds, with higher thresholds indicating a stronger volumetric asymmetry in a given voxel. These results are depicted in Fig. 3D. For both datasets, the *t* test shows comparably high similarity with LQ maps at lower thresholds, but with a progressive decrease of similarity with increasing thresholds, which holds true for both leftward and rightward laterality quotients. For dataset 1, the highest similarity between *t* test results and LQ were found at a threshold of 0.04 for both positive (DSC = 0.437) and negative LQs (DSC = 0.55). In comparison, the map gained from Boruta selection shows little resemblance with the laterality quotient at lower thresholds, but increases with higher LQs in both datasets, regardless of the LQ direction. For dataset 1, the similarity between Boruta results and LQ maps peaked for positive LQ (rightward) at a threshold of 0.22 (DSC = 0.118) and for negative LQ (leftward) at a threshold of − 0.16 (DSC = 0.194). For dataset 2, the similarity between Boruta results and LQ maps peaked for positive LQ (rightward) at a threshold of 0.22 (DSC = 0.107) and for negative LQ (leftward) at a threshold of − 0.14 (DSC = 0.218). The full list of dice similarity coefficients can be found in the supplementary material (table S1, table S2).

### Hemisphere classifiability across sex and handedness

**Fig. 4:**
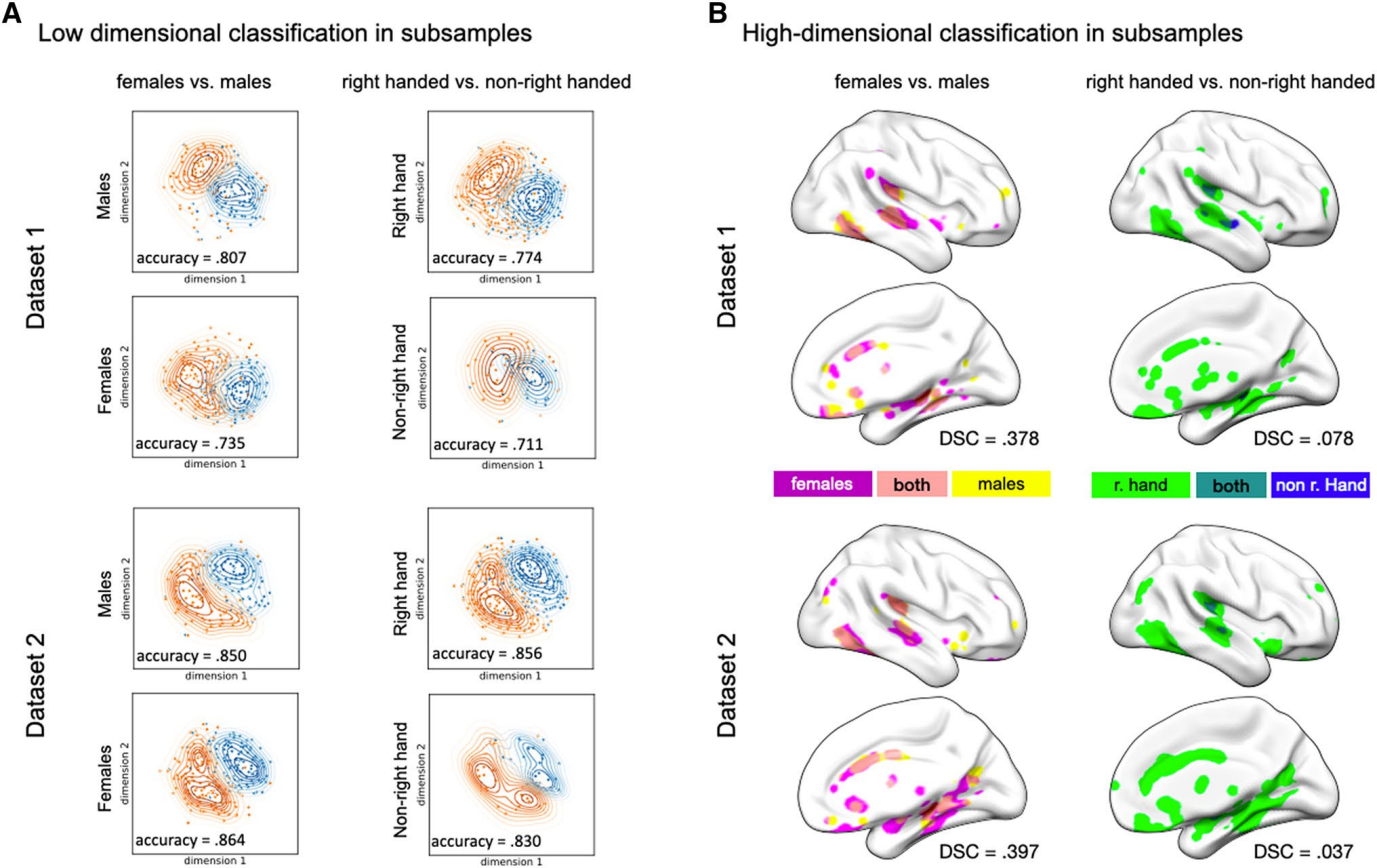
Comparing hemisphere classifiability between subsamples. **A** Low-dimensional classification in females vs. males (left column) and right-handed vs. non-right-handed participants (right column). Each panel depicts a KDE-plot with an embedded scatterplot, in which both the left (blue) and right (orange) hemispheres are depicted in their low-dimensional representation. The first dimension is always depicted on the x-axis. The reported accuracy values represent the averaged SVM based cross-validated accuracy for identifying the side of a given hemisphere. **B** Boruta selection. The left column depicts the comparison between females (light green) and males (blue) as well as their overlap (dark green). The right column depicts the comparison between right-handed (light green) and non-right-handed participants (blue) as well as their overlap (dark green). The dice similarity coefficient (DSC) represents the overlap between voxels in females and males, or right and non-right-handed participants, which contributed to correctly classifying the hemispheres as either left or right. For both subfigures, the results of dataset 1 are depicted in the upper part and results of dataset 2 are shown in the lower part of the figure

Sex and handedness are traits that are associated with hemispheric asymmetries. We, therefore, split the two samples with regard to either sex or handedness, to partially evaluate the effect of these phenotypes on the hemisphere classifiability on both low- and high-dimensional spaces. The results of both classification approaches are depicted in Fig. 4.

For the low-dimensional classification (Fig. 4A), the dimensionality of hemispheres was again reduced to two via UMAP and the classifiability of hemispheres was assessed via SVM for each subsample. The results indicate that SVM was capable of differentiating the hemispheres in each group, given that classification accuracy was well above chance level. In dataset 1, the hemispheres of males showed a higher classifiability (accuracy = 0.807) than the ones of females (accuracy = 0.735). In contrast, the opposite was found in dataset 2, in which the hemispheres were more accurately classified in females (accuracy = 0.864) compared to males (accuracy = 0.850). With regard to handedness, hemispheres of right-handed participants were more accurately classified compared to the ones of non-right-handed participants, irrespective of the dataset (dataset 1: right-handed = 0.774, non-right-handed = 0.771; dataset 2: right-handed = 0.856, non-right-handed = 0.830).

The accuracy of voxel-based hemisphere classification was assessed using a LASSO classifier for each subsample (males, females, right-handed participants and non-right-handed participants). The results indicated high averaged accuracy for identifying the side of a given hemisphere. For dataset 1, classification accuracy was above 90% for females (accuracy = 0.957) and males (accuracy = 0.948), as well as for right-handed participants (accuracy = 0.974), but comparably lower for non-right-handed participants (accuracy = 0.821). For dataset 2, the same pattern occurred with near perfect hemisphere classification for females (accuracy = 0.964), males (accuracy = 0.925), and right-handed participants (accuracy = 0.953), compared to a relatively lower classification accuracy in non-right-handed participants (accuracy = 0.853). The overlap of Boruta-selected voxels was assessed via the DSC. The pattern of results was comparable across the two datasets. Females and males showed a good overlap of Boruta-selected voxels (DSC dataset 1 = 0.378; DSC dataset 2 = 0.397), with best overlap between the two sexes showing in the planum temporale, cingulate gyrus, parietal operculum, parahippocampal gyrus, hippocampus, lateral occipital cortex and the frontal medial cortex. Comparing the Boruta-selected voxels between the right-handed and non-right-handed participants, however, only showed a very small overlap (DSC dataset 1 = 0.078; DSC dataset 2 = 0.037) which was accompanied by a large distribution of informative voxels in right-handed participants, but only very sparse clusters of voxels shown in the other group. Concordantly, the overlap between these groups is seen in the hippocampus, middle temporal gyrus, parietal operculum, and the planum temporale (Kong et al. 2018; Guadalupe et al. 2017).

## Discussion

Past studies identified several asymmetries between the two hemispheres, but the defining characteristics that distinguish the two hemispheres are yet to be determined. While conventional statistical approaches are best designed to find asymmetries between the hemispheres, they are not designed for finding the features that are necessary to distinguish the hemispheres from one another. Therefore, we introduced a novel framework for investigating hemispheric differences by not focusing on significant asymmetries, but instead asking the question of which information can be used to accurately classify the left and right hemisphere. Machine learning-based classification was used to distinguish left from right hemispheres, on the basis of both their low- and high-dimensional representations. As a proof-of-concept, classifiers were fed with voxel-based morphometry data and all analyses were performed in two different samples. Independent of the sample, the classification results evinced high separability of the two hemispheres based on volumetric maps. Hence, our study supports the utility of a classification framework for investigating hemispheric differences and limiting the search space of the two hemispheres’ defining characteristics.

For the low-dimensional classification approach, hemispheres were separated and embedded into a low-dimensional space using manifold learning. Here, hemispheres that are more similar to each other will be embedded in near proximity and hemispheres that are dissimilar will be embedded more distantly from each other. The high classification accuracy of a subsequently applied support vector machine demonstrated that the two hemispheres were distinguishable based on global properties which are preserved in their low-dimensional representation. This indicates that the proposed low-dimensional classification framework should be applicable independently of the chosen brain representation (such as summary metrics of graph-theory analyses or gradient approaches). This is particularly useful, given that dimensionality reduction via manifold learning techniques appears to be capable of unraveling organization principles of the brain. For instance, the hierarchical topology of the functional brain architecture is observable based on the principal gradient that spans from sensorimotor to transmodal areas (Margulies et al. 2016). In addition, other cortical features such as myelination (Huntenburg et al. 2017) or the representation of event length (Baldassano et al. 2017) share this topological axis (Huntenburg et al. 2018). In addition, organizational axes uncovered by manifold learning have been linked to evolutionary principles such as the progressive differentiation of cortical layers (Waymel et al. 2020; Valk et al. 20) as proposed by the dual-origin theory (Dart, 1934; Pandya et al. 2015). Therefore, manifold learning techniques provide useful insights into the topology of the human brain, both on a local/regional scale as well as on a global/whole-brain level. With regard to brain asymmetries, a recent study indicates a leftward asymmetry in the range of the principal gradient (Liang et al. 2021). This asymmetry is influenced by sex, indicated by a larger leftward asymmetry in males, which is in accordance with typical sex differences in brain asymmetries (Hirnstein et al. 2019; Sommer et al. 2008). However, the overall topology of the principal gradient is mostly symmetrical (Liang et al. 2021), thus indicating that resting-state functional connectivity profiles of left- and right-hemispheric cortical regions may be similar in terms of their hierarchical organization. Therefore, it is yet to be seen if the principal gradient distinguishes the two hemispheres. This is one exemplary question that can be addressed by future studies using the classification framework. In addition to the overall classification accuracy, the low-dimensional approach indicated a hemispheric difference in the classifiability, with higher precision for classifying the right hemispheres compared to the left hemispheres. Given that variability positively impacts classifier performance (Therrien and Doyle 2018), the higher variance of the right-hemispheric low-dimensional representations may play a role for this difference. However, it is not yet clear why the variance of the manifold of the right hemisphere is higher in the first place. Further investigations will be needed to shed light onto this topic as more studies may utilize embedding approaches for projecting the complex and high-dimensional data structure of each hemisphere into lower dimensions.

For the high-dimensional classification approach, volumetry of all voxels from each single hemisphere were used as input features of the classifier. For the high-dimensional classification approach, volumetry of all voxels from each single hemisphere were used as input features of the classifier. A LASSO classifier showed very high accuracies for classifying the hemispheres as either left or right in both datasets. Subsequent usage of the Boruta feature selection method revealed voxels that contributed to the classification. These voxels reside mostly in brain regions that have been reported to show asymmetries (e.g., Kong et al. 2018, Chiarello et al. 2016). With respect to the input data, this approach is akin to a voxel-wise comparison between the two hemispheres using univariate statistics. Hence, we computed one-sample *t* tests on the laterality quotient maps (which is equivalent to paired sample *t* tests on the left vs. right hemispheres), to compare results of the two approaches. While there is some visual overlap between the two methods, especially with regard to prominently asymmetric regions, the evaluation of their respective similarity with the laterality quotient maps revealed differences with increasing thresholds. Here, the *t* test results showed strong similarities with the distribution of negative and positive LQ values, at lower thresholds, which decreased with more strongly lateralized voxels. In comparison, the map of contributing voxels was better at representing voxels with middle and higher LQ values, regardless of the direction of their lateralization. Given that the advantage in representing LQ values shifts from *t* tests to Boruta selection, these two methods appear complementary to one another.

While the main goal of this investigation was to present a proof-of-concept for the classification approach in both low- and high-dimensional data, we additionally investigated hemisphere classifiability for separate participant groups including males and females as well as right-handed and non-right-handed participants.

While the results of the SVM classifier indicated that the two hemispheres can be distinguished in their low-dimensional representation in each subsample, the averaged classification accuracies displayed a strong effect of the dataset on the result pattern: on the one hand, the classification accuracies were generally higher in dataset 2, compared to dataset 1. On the other hand, comparing the accuracy between males and females shows different outcomes, with better accuracy for males in dataset 1, but better accuracy of females in dataset 2. As described in Snoek et al. (2021), t1-weighted images from the two datasets differed in their contrast-to-noise ratio. Therefore, it can be assumed that UMAP embedding might be highly affected to the contrast-to-noise ratio, thus subsequently affecting the accuracies for hemisphere classification, which will need to be tested in future studies by varying contrast-to-noise ratio in the same participants.

In contrast to the low-dimensional classification, the high-dimensional classification via LASSO and Boruta indicated a good fit of the results gained from the two datasets. Comparing the accuracy between the groups indicated that hemispheres from males, females, and right-handed participants (irrespective of sex) were highly classifiable with accuracies well above 90%, whereas the accuracy for classifying hemispheres was below 90% in the non-right-handed subsamples. Importantly, participants acquired in the used datasets were not specifically selected with regard to their handedness. This led to relatively lower numbers of non-right-handed participants (dataset 1 = 29, dataset 2 = 23) compared to the group of right-handed participants (dataset 1 = 180; dataset 2 = 210). The similarity of the map of Boruta-selected voxels between males and females indicated good overlap especially in regions with reported asymmetries such as the planum temporale, middle temporal gyrus and the hippocampus (Kong et al. 2018; Guadalupe et al. 2017). Comparing the maps of Boruta-selected voxels between the right-handed and non-right-handed subsample showed only small clusters of overlapping voxels. This overlap was mostly found in the hippocampus and planum temporale, which highlights their relevance for classifying hemispheres. The planum temporale is of particular interest for laterality research, given its accentuated role in the lateralization of speech perception (Moffat et al. 1998; Ocklenburg et al. 2018). Furthermore, the leftward asymmetry of the planum temporale has been robustly documented based on imaging studies (Kong et al. 2018) as well as from histological analyses (Geschwind and Levitsky 1968; Galaburda et al. 1978). Likewise, the hippocampus is rightward asymmetric in adults (Pedraza et al. 2004; Guadalupe et al. 2017) as well as in infants (Thompson et al. 2009). While the relevance of hippocampal asymmetries is still a matter of investigation, there is evidence for functional lateralization of the hippocampus, indicated by more severe spatial memory deficits in patients with right-hemispheric hippocampal lesions compared to left hippocampal patients (Kessels et al. 2001).

As our approaches for mapping differences between the two hemispheres become more diverse, a methodological framework designed to investigate determining characteristics of the two hemispheres must be applicable across different brain representations and irrespective of dimensionality. Our study indicates that assessing the classifiability of each hemisphere promises to be a strong candidate for shedding light onto the determining features of each hemisphere. Similarly, machine learning-based classification has proven to be a useful addition to the modern neuroscientist’s methodological repertoire for a variety of research questions. Examples range from more basic neuroscientific applications such as sex classification based on resting-state connectivity (Weis et al. 2020) or gray matter anatomy (Anderson et al. 2019), to more applied questions including diagnostic classification of psychiatric or neurological patients (Yassin et al. 2020; Yassin et al. 2020; Klöppel et al. 2008) or even the classification of endophenotypes of functional impairment caused by brain lesions (Rehme et al. 2015). Consequently, studies that promote the application of data-driven and machine learning-based methods that are tailored towards studying brain asymmetries may grant new insights in this field (Ocklenburg et al. 2020).

### Limitations and outlook

While the pattern of results indicates the feasibility of the classification framework to (a) investigate if a low-dimensional representation allows to classify the two hemispheres, and (b) to classify the hemispheres in their high-dimensional representation and identify brain units (here voxels) that allow to distinguish between the two hemispheres, the present study comes with a set of choices and limitations.

For the low-dimensional classification, we chose to reduce dimensionality using UMAP, as it is particularly suited for revealing underlying organization patterns of high-dimensional data especially with very high number of observations. For example, UMAP has been rapidly adopted in the field of population genetics (Diaz-Papkovich 2021), successfully applied to visualize single-cell RNA sequencing (Becht et al. 2019), investigate phenotype heterogeneity across genetic cohorts (Diaz-Papkovich 2019), or to reveal shared population structure of modern and ancient human DNA (Margaryen et al. 2020). In neuroimaging, UMAP has been used for distinct tasks such as to gain information about the general relation between different analyses approaches (Dafflon et al. 2020), display similarity in white matter tractography results between research groups (Schilling et al. 2020), segment the corpus callosum based on functional hierarchy (Friedrich et al. 2020), as well as to investigate the difference between syntactic and real brain lesions (de Schotten et al. 2020). Therefore, using dimensionality reduction via UMAP appears to be a reasonable methodological choice for creating low-dimensional representations of the hemispheres.

With regard to the high-dimensional classification, one important limitation is the lack of directionality in the selected features (voxels). The feature selection algorithm only identifies voxels that correctly distinguish the two hemispheres. Although it is plausible to assume that these voxels represent determining characteristics of each hemisphere, the approach does not tell for which one of the two hemispheres is characterized by these voxels. Therefore, we interpret the selected features as candidates for inhabiting characteristic features with regard to the metric or summary measure that is represented within the voxel.

In cognitive neuroscience, machine learning is usually used to predict phenotypes or labels such as age, mental health status, sex or personality from neuroimaging data. As the focus lies on predicting phenotypes in unknown samples, a supervised algorithm typically learns the relation between input features and the target value in a training dataset. Subsequently, the performance of the trained algorithm is tested on an unknown test dataset, which indicates the generalizability of the algorithm. In the current study, however, we did not test the generalizability of the classifier, as the primary aim of this framework is not to correctly predict the side of unknown hemispheres, but rather to identify the features that are capable of distinguishing the hemispheres by means on the summary measure of interest. Therefore, we do not assume the classifiers to validly predict hemispheres outside of the tested samples due to potential overfitting, despite the use of cross-validation.

The current study serves as a proof-of-concept for validating the ability of machine learning-based classification to distinguish between the two hemispheres both in their low- and high-dimensional representations, as well as assessing the possibility to restrict the search for defining features of each hemisphere. We, therefore, did not choose ideal datasets for comparing the hemisphere classifiability across sex and handedness. In addition, the age range of participants in both datasets was rather limited. As age plays an important role for brain asymmetries and lateralized cognition (Kovalek et al. 2003), further investigations are called for that focus on addressing hemisphere classifiability across participants with various traits.

In this study, we only focused on volumetric data due to the large body of literature that indicates—albeit with some inconsistencies—the presence of brain asymmetries in volumetry. Our results suggest the classification approach to be feasible for differentiating the hemispheres in both their low-dimensional and high-dimensional volumetric representation. Furthermore, feature selection was able to locate the voxels that are meaningful for the classifiability of hemispheres, which represents a new approach for mapping relevant hemispheric differences. Accessing the classifiability of the two hemispheres with other metrics of interest that have been shown asymmetries, such as neurite density (Schmitz et al. 2019); myelination (Ocklenburg et al. 2019; O'Muircheartaigh et al. 2013) or white matter integrity (Büchel et al. 2004) in both healthy participants and patient cohorts, will be an important matter of interest for future research. In this regard, it is worth noting that the proposed classification framework is not limited to only include information from one structural or functional measure. Information from multiple imaging modalities and representations can be combined, which will result in increased initial dimensionality, and in turn higher computational demands. Studies that aim to characterize the hemispheres from a multimodal perspective may grant a wider picture on the question about defining characteristics of each hemisphere.

## Conclusion

In spite of the multi-methodological perspectives and numerous studies on hemispheric asymmetries, we are yet to understand the defining characteristics of each hemisphere. In this work, we introduce hemisphere classifiability as a framework for investigating features that distinguish the left and right hemispheres. Our study shows that the two hemispheres are classifiable based on volumetric information both in their high- and low-dimensional representations. The high-dimensional approach revealed a set of voxels that allows distinguishing the hemispheres, whereas the low-dimensional approach indicates that the approximated topology of hemispheres is more similar between hemispheres from the same side. The classification framework is scalable and universally applicable across brain representations, thus allowing for the characterization of the hemispheres from a multimodal perspective. As advances in neuroscience are typically driven by the invention and novel application of research methods (Greenwald 2012; Yuste 2015), we hope to draw nearer to a more complete characterization of the two sides of our brains.

## Supplementary Information

Below is the link to the electronic supplementary material.

Supplementary file1 (DOCX 18 KB)

Supplementary file2 (DOCX 19 KB)

## Data Availability

All data in this study are openly available (https://nilab-uva.github.io/AOMIC.github.io/). All code used in this study can be shared on request.
