# Supplementary material for: Is it left or is it right? A classification approach for investigating hemispheric differences in low and high dimensionality
Source: Brain Struct Funct. 2021 Dec 9;227(2):425–40. doi: 10.1007/s00429-021-02418-1 (PMC8844166; doi:10.1007/s00429-021-02418-1)
Supplement: Supplementary file 1 — Supplementary file1 (DOCX 18 KB) [file 429_2021_2418_MOESM1_ESM.docx]

**Table S1 – Dice similarity coefficient for Dataset 1**

| **method** | **thresholds** | **LQall** | **LQpos** | **LQneg** |  | **method** | **thresholds** | **LQall** | **LQpos** | **LQneg** |
| --- | --- | --- | --- | --- | --- | --- | --- | --- | --- | --- |
| boruta | 0.02 | 0.028184 | 0.023825 | 0.031339 |  | tfce | 0.02 | 0.612593 | 0.406087 | 0.521898 |
| boruta | 0.04 | 0.041227 | 0.033855 | 0.046181 |  | tfce | 0.04 | 0.698362 | 0.437220 | 0.551159 |
| boruta | 0.06 | 0.062364 | 0.048584 | 0.071103 |  | tfce | 0.06 | 0.675647 | 0.403028 | 0.483633 |
| boruta | 0.08 | 0.089171 | 0.066646 | 0.101463 |  | tfce | 0.08 | 0.556719 | 0.311419 | 0.366709 |
| boruta | 0.10 | 0.123525 | 0.084559 | 0.142150 |  | tfce | 0.10 | 0.423316 | 0.225162 | 0.260153 |
| boruta | 0.12 | 0.158479 | 0.101385 | 0.178571 |  | tfce | 0.12 | 0.306613 | 0.156892 | 0.179393 |
| boruta | 0.14 | 0.177850 | 0.111283 | 0.187819 |  | tfce | 0.14 | 0.218535 | 0.109901 | 0.122636 |
| boruta | 0.16 | 0.193190 | 0.113162 | 0.193833 |  | tfce | 0.16 | 0.152839 | 0.077191 | 0.082174 |
| boruta | 0.18 | 0.195089 | 0.116959 | 0.174807 |  | tfce | 0.18 | 0.107404 | 0.057069 | 0.053413 |
| boruta | 0.20 | 0.197783 | 0.117174 | 0.159607 |  | tfce | 0.20 | 0.075420 | 0.041267 | 0.035626 |
| boruta | 0.22 | 0.205656 | 0.118265 | 0.152039 |  | tfce | 0.22 | 0.053767 | 0.029225 | 0.025278 |
| boruta | 0.24 | 0.199403 | 0.111828 | 0.136503 |  | tfce | 0.24 | 0.038492 | 0.022078 | 0.016784 |
| boruta | 0.26 | 0.192204 | 0.102884 | 0.125305 |  | tfce | 0.26 | 0.027640 | 0.015470 | 0.012360 |
| boruta | 0.28 | 0.177515 | 0.088333 | 0.113946 |  | tfce | 0.28 | 0.019703 | 0.010621 | 0.009179 |
| boruta | 0.30 | 0.164800 | 0.078947 | 0.102293 |  | tfce | 0.30 | 0.013618 | 0.007013 | 0.006651 |
| boruta | 0.32 | 0.139883 | 0.068284 | 0.082734 |  | tfce | 0.32 | 0.010681 | 0.005385 | 0.005325 |
| boruta | 0.34 | 0.119896 | 0.053555 | 0.073260 |  | tfce | 0.34 | 0.007675 | 0.003573 | 0.004117 |
| boruta | 0.36 | 0.101514 | 0.043071 | 0.063021 |  | tfce | 0.36 | 0.005988 | 0.002666 | 0.003331 |
| boruta | 0.38 | 0.085145 | 0.030246 | 0.057944 |  | tfce | 0.38 | 0.004842 | 0.002061 | 0.002787 |
| boruta | 0.40 | 0.068203 | 0.019084 | 0.050895 |  | tfce | 0.40 | 0.003694 | 0.001455 | 0.002242 |
| boruta | 0.42 | 0.058052 | 0.011572 | 0.047393 |  | tfce | 0.42 | 0.002666 | 0.000788 | 0.001879 |
| boruta | 0.44 | 0.050943 | 0.007752 | 0.043726 |  | tfce | 0.44 | 0.002182 | 0.000485 | 0.001697 |
| boruta | 0.46 | 0.038059 | 0.005825 | 0.032536 |  | tfce | 0.46 | 0.001637 | 0.000364 | 0.001273 |
| boruta | 0.48 | 0.036364 | 0.005831 | 0.030769 |  | tfce | 0.48 | 0.001273 | 0.000303 | 0.000970 |
| boruta | 0.50 | 0.025048 | 0.001949 | 0.023166 |  | tfce | 0.50 | 0.000849 | 0.000121 | 0.000728 |
| boruta | 0.52 | 0.017408 | 0.001949 | 0.015504 |  | tfce | 0.52 | 0.000607 | 0.000121 | 0.000485 |
| boruta | 0.54 | 0.011650 | 0.000000 | 0.011650 |  | tfce | 0.54 | 0.000364 | 0.000000 | 0.000364 |
| boruta | 0.56 | 0.011650 | 0.000000 | 0.011650 |  | tfce | 0.56 | 0.000364 | 0.000000 | 0.000364 |
| boruta | 0.58 | 0.005842 | 0.000000 | 0.005842 |  | tfce | 0.58 | 0.000182 | 0.000000 | 0.000182 |
| boruta | 0.60 | 0.005842 | 0.000000 | 0.005842 |  | tfce | 0.60 | 0.000182 | 0.000000 | 0.000182 |
