# Supplementary material for: Is it left or is it right? A classification approach for investigating hemispheric differences in low and high dimensionality
Source: Brain Struct Funct. 2021 Dec 9;227(2):425–40. doi: 10.1007/s00429-021-02418-1 (PMC8844166; doi:10.1007/s00429-021-02418-1)
Supplement: Supplementary file 2 — Supplementary file2 (DOCX 19 KB) [file 429_2021_2418_MOESM2_ESM.docx]

**Table S2 – Dice similarity coefficient for Dataset 2**

| **method** | **thresholds** | **LQall** | **LQpos** | **LQneg** |  | **method** | **thresholds** | **LQall** | **LQpos** | **LQneg** |
| --- | --- | --- | --- | --- | --- | --- | --- | --- | --- | --- |
| boruta | 0.02 | 0.039815 | 0.035485 | 0.041959 |  | tfce | 0.02 | 0.696247 | 0.406168 | 0.608647 |
| boruta | 0.04 | 0.054717 | 0.045737 | 0.059210 |  | tfce | 0.04 | 0.759908 | 0.419461 | 0.621146 |
| boruta | 0.06 | 0.075600 | 0.056639 | 0.085717 |  | tfce | 0.06 | 0.708794 | 0.379841 | 0.530346 |
| boruta | 0.08 | 0.099125 | 0.067940 | 0.114796 |  | tfce | 0.08 | 0.573383 | 0.298096 | 0.393212 |
| boruta | 0.10 | 0.124894 | 0.075829 | 0.148383 |  | tfce | 0.10 | 0.435474 | 0.221979 | 0.276146 |
| boruta | 0.12 | 0.155881 | 0.083878 | 0.186175 |  | tfce | 0.12 | 0.319882 | 0.160650 | 0.190843 |
| boruta | 0.14 | 0.185953 | 0.090765 | 0.218263 |  | tfce | 0.14 | 0.231191 | 0.117216 | 0.129609 |
| boruta | 0.16 | 0.193267 | 0.095586 | 0.210803 |  | tfce | 0.16 | 0.166563 | 0.087195 | 0.087137 |
| boruta | 0.18 | 0.189010 | 0.096538 | 0.187800 |  | tfce | 0.18 | 0.119556 | 0.066766 | 0.056617 |
| boruta | 0.20 | 0.183813 | 0.101483 | 0.156658 |  | tfce | 0.20 | 0.085189 | 0.048626 | 0.038448 |
| boruta | 0.22 | 0.187097 | 0.106714 | 0.139053 |  | tfce | 0.22 | 0.060147 | 0.035233 | 0.025831 |
| boruta | 0.24 | 0.185989 | 0.103013 | 0.129412 |  | tfce | 0.24 | 0.044663 | 0.026727 | 0.018431 |
| boruta | 0.26 | 0.179630 | 0.099738 | 0.113662 |  | tfce | 0.26 | 0.031994 | 0.019962 | 0.012277 |
| boruta | 0.28 | 0.165824 | 0.090200 | 0.099461 |  | tfce | 0.28 | 0.023594 | 0.014926 | 0.008799 |
| boruta | 0.30 | 0.144816 | 0.080331 | 0.079159 |  | tfce | 0.30 | 0.016176 | 0.009960 | 0.006279 |
| boruta | 0.32 | 0.126012 | 0.070689 | 0.064721 |  | tfce | 0.32 | 0.011747 | 0.007491 | 0.004288 |
| boruta | 0.34 | 0.101205 | 0.050346 | 0.056483 |  | tfce | 0.34 | 0.008412 | 0.004968 | 0.003461 |
| boruta | 0.36 | 0.085502 | 0.037347 | 0.051680 |  | tfce | 0.36 | 0.006182 | 0.003217 | 0.002974 |
| boruta | 0.38 | 0.067216 | 0.027469 | 0.041694 |  | tfce | 0.38 | 0.004385 | 0.002049 | 0.002341 |
| boruta | 0.40 | 0.054019 | 0.018457 | 0.036721 |  | tfce | 0.40 | 0.003315 | 0.001464 | 0.001854 |
| boruta | 0.42 | 0.043053 | 0.011968 | 0.031662 |  | tfce | 0.42 | 0.002244 | 0.000830 | 0.001415 |
| boruta | 0.44 | 0.034166 | 0.010667 | 0.023857 |  | tfce | 0.44 | 0.001708 | 0.000635 | 0.001074 |
| boruta | 0.46 | 0.027723 | 0.006680 | 0.021262 |  | tfce | 0.46 | 0.001366 | 0.000488 | 0.000879 |
| boruta | 0.48 | 0.022561 | 0.004024 | 0.018629 |  | tfce | 0.48 | 0.000976 | 0.000195 | 0.000781 |
| boruta | 0.50 | 0.021262 | 0.004024 | 0.017322 |  | tfce | 0.50 | 0.000879 | 0.000195 | 0.000683 |
| boruta | 0.52 | 0.016000 | 0.002686 | 0.013351 |  | tfce | 0.52 | 0.000635 | 0.000098 | 0.000537 |
| boruta | 0.54 | 0.012024 | 0.002686 | 0.009365 |  | tfce | 0.54 | 0.000488 | 0.000098 | 0.000391 |
| boruta | 0.56 | 0.010695 | 0.001344 | 0.009365 |  | tfce | 0.56 | 0.000439 | 0.000049 | 0.000391 |
| boruta | 0.58 | 0.008038 | 0.000000 | 0.008038 |  | tfce | 0.58 | 0.000293 | 0.000000 | 0.000293 |
| boruta | 0.60 | 0.005366 | 0.000000 | 0.005366 |  | tfce | 0.60 | 0.000195 | 0.000000 | 0.000195 |
